# The Baby Triple P online positive parenting programme for mothers accessing community perinatal mental health care (the OPAL study): a feasibility study protocol

**DOI:** 10.3389/fpsyt.2026.1766060

**Published:** 2026-03-03

**Authors:** Anja Wittkowski, Henna Lemetyinen, Holly E. Reid, Trinity Perruzza-Powell, Lynsey Gregg

**Affiliations:** 1 Division of Psychology and Mental Health, School of Health Sciences, Faculty of Biology, Medicine and Health, The University of Manchester, Manchester, United Kingdom; 2 The Perinatal Mental Health and Parenting (PRIME) Research Unit, Greater Manchester Mental Health National Health Service (NHS) Foundation Trust, Manchester, United Kingdom; 3 Manchester Academic Health Sciences Centre, Manchester, United Kingdom

**Keywords:** intervention, mothers, parenting, parents, severe mental illness, women

## Abstract

**Background:**

Perinatal mental health difficulties can negatively impact maternal wellbeing, which in turn can impact the mother-infant bond. Although parenting interventions have been found to be effective, they are often not routinely offered in mental health services as additional psychosocial support. Thus, the aims of this study are a) to examine the feasibility of recruiting mothers, engaging them in an online parenting intervention and retaining them in the study and b) to explore the acceptability of this type of intervention in mothers and specialist perinatal mental health staff. We will also explore any changes in relevant outcomes for mothers.

**Methods:**

In this uncontrolled feasibility study, women experiencing moderate to severe mental health problems, who are in the later of stage of their pregnancy or mother to a baby up to 12 months old, will be recruited from a perinatal community mental health service in the Northwest of England, UK. Consented participants will be offered the self-paced Triple P for Baby Online parenting intervention alongside any service support. To support engagement, the research team will offer four check-in phone calls and text messages to support participants. Outcomes measures, completed online at baseline and again at post-intervention ten weeks after study enrolment, will examine changes in maternal mental health (depression, anxiety, stress), wellbeing, maternal self-efficacy and the perceived mother-infant bond.

**Results:**

Descriptive summaries will be produced for feasibility outcomes, including recruitment and retention rates and number of sessions completed. We will use paired t-test or Wilcoxon signed rank test for pre- and post-intervention questionnaire data and the reliable change index to explore any changes in outcomes. Mothers (n=10-20) and staff (n=5-10) will be interviewed to explore acceptability, engagement and implementation factors. Interview data will be analysed using framework analysis.

**Discussion:**

This is the first study to examine the feasibility of engaging women in a self-paced, online parenting intervention and its acceptability within a NHS perinatal community mental health service. We will also explore its potential benefits in terms of outcomes. If progression to a full trial is indicated, this study will inform future study design, recruitment methods, eligibility criteria and outcome measures.

**Trial registration:**

Prospective ISRCTN registration (11/03/2025): ISRCTN87365121; https://doi.org/10.1186/ISRCTN87365121.

## 1 Introduction

Parenting interventions have consistently demonstrated strong effectiveness and efficacy in promoting positive developmental outcomes for both parents and children, with benefits ranging from improved parenting skills and reduced child behavioural problems to overall enhanced family functioning (1, 2). Among these interventions, the Triple P–Positive Parenting Program stands out as one of the most extensively researched, evidence-based, and scalable frameworks (3–7). Grounded in social learning theory, cognitive-behavioural, and developmental psychology, Triple P has been shown to reduce coercive parenting, increase parental self-efficacy, and significantly decrease child conduct problems across diverse populations and cultural contexts (3, 4, 7–11). Meta-analytic evidence further indicates that parenting programmes like Triple P foster long-term gains in child emotional regulation, decrease parental stress, and strengthen parent–child relationships, highlighting their value as both preventative and therapeutic tools (3, 4, 7, 8, 12, 13). Collectively, structured, evidence-based parenting interventions play a critical role in supporting family resilience, promoting healthy child development, and reducing the burden of mental health and behavioural difficulties across communities. Although the majority of evidence focuses on parenting programmes for preschool and school-aged children, similar principles of early relational and psychoeducational support underpin interventions designed for infants.

The transition to parenthood represents a major developmental milestone characterised by profound psychological, relational, and practical adjustments (14, 15). New parents frequently experience elevated stress, sleep disruption, and changes in couple functioning, all of which heighten vulnerability to perinatal mental health difficulties, such as depression and anxiety (16, 17). Parental mental health is closely intertwined with the quality of early caregiving behaviours (18, 19). Parental distress can undermine sensitive and responsive caregiving, whereas supporting parents to feel competent, regulated, and emotionally available strengthens the emerging parent–infant bond (20–22). This bond forms the foundation for infants’ emotional regulation, attachment security, and later socio-emotional development (15, 23–25). As interventions that support and strengthen parental wellbeing alongside early relationship-building are vital for fostering healthy developmental trajectories in both parent and child (26), there has been an increased focus by governments, especially in the United Kingdom (UK), to make the provision of early intervention, enhanced perinatal mental health services and support for vulnerable families a national priority (e.g., 27–29).

Triple P for Baby was developed to support new parents (e.g., mothers, fathers, etc.) transitioning to parenthood by targeting known modifiable risk and protective factors (30–32). It promotes realistic expectations about parenthood, the couple’s changing relationship, infant behaviour and development, whilst offering parenting skills training to increase parenting competence and adaptive strategies for emotion regulation. Through Triple P’s underlying self-sufficiency model (3–5, 13) Triple P for Baby also seeks to equip parents with knowledge and skills as well as the confidence to independently manage common parenting challenges. It promotes the development of a strong parent-infant bond, the strengthening of the parents’ coping resources and the improvement of both parents supporting each other whilst drawing on wider social support (30–32).

Triple P for Baby was originally designed as a four-session antenatal group intervention, followed by four postnatal individual telephone support sessions, but it has been delivered individually and postnatally only as well. Triple P for Baby’s acceptability and feasibility and potential benefits have been demonstrated in various studies including randomised controlled trials with primiparous and multiparous parents in diverse settings (e.g., 31–44). For example, a larger UK RCT involving first-time parent couples demonstrated strong compliance and engagement with the programme, indicating its practical acceptability (38). However, a follow-up sensitivity analysis of a sub-sample of data revealed limited effects on the couple relationship, social support and parenting skills. But caution should be exercised in the interpretation of these findings because the sub-sample was underpowered (38). Furthermore, a large UK RCT comparing an enhanced version of Triple P for Baby and Mellow Bumps against standard care in a group of women with complex social and care needs did not demonstrate efficacy for any parenting intervention in improving mental health and wellbeing outcomes for women in this context of heightened social complexity (37).

Despite being a universal parenting intervention, Triple P for Baby has also been explored as an additional treatment for women with mental health problems; for example, in a pilot RCT with mothers experiencing postnatal depression reported high levels of participant engagement and satisfaction (42). Furthermore, a multi-site feasibility RCT evaluated the individually and postnatally delivered intervention in a perinatal mental health setting (psychiatric Mother and Baby Unit) and reported that 75% of mothers completed the intervention, which these mothers also rated as highly acceptable (44). Clinical outcomes signalled potential improvements in parenting competence, bonding, mood and mental health symptomatology, but a full trial in a specialist perinatal mental health setting in the UK has yet to be undertaken. Finally, a full RCT in an urban Kenyan setting, with Afrikan mothers experiencing depression and anxiety, showed improvements in maternal mental health outcomes, but no significant difference was found in maternal self-efficacy between the mothers who did and those who did not access Triple P for Baby (33).

The COVID-pandemic in 2020 accelerated the development of online interventions including a seven-session, self-paced, self-guided, interactive version of Triple P for Baby Online. It is highly accessible (via laptop, tablet or phone) to parents of infants, minimising the need for external childcare to attend the intervention in person and, given its interactive content with brief video-clips, it is also suitable for people with different literacy and educational levels. Online versions of Triple P have been shown to be as effective as other modalities in general population samples (3, 4, 45). In 2022, a national rollout of Triple P Online took place across Australia which allowed all families with children under 12 years of age across the country free access to Triple P’s evidence-based suite of online programmes, including the intervention for parents of babies (46). Similarly, the strength of Triple P for Baby’s existing evidence and its ability to support parental wellbeing and the parent-infant-relationship led the Department of Health and Social Care and NHS England in 2021 to commission the training of 680 practitioners in this intervention as part of the *Start for Life/Family Hub* offer for 75 local authorities (47). Many local authorities also commissioned the delivery of Triple P for Baby Online to address the Department of Work and Pensions’ Reducing Parental Conflict Programme (48) and the *Supporting Families Outcome Framework* (49).

With an increasing emphasis on digital approaches to improving mental health care, the proposed project allows us to explore the feasibility and acceptability of this parenting intervention whose online version has not yet been tested in a perinatal mental health context. Triple P for Baby Online is an ideal supplementary intervention to strengthen parenting competence, the parent-infant bond and partner support – important factors which are not always targeted by perinatal mental health services.

Up to 27% of women experience maternal mental illness in the perinatal period (50), with maternal suicide being the leading cause of death for women between six weeks and one year after pregnancy in the UK (51). In the UK, perinatal mental health services have been expanded to improve access to assessment and treatment for women as a public health priority (29, 52, 53). This increased access is expected to minimise the risks that maternal mental illness can pose for the development of bonding and attachment difficulties between mother and baby and any subsequent negative impacts on the child’s development (18, 54, 55).

The type and severity of the woman’s mental health problem in the perinatal period will determine the type of intervention offered (56). In England, 81,975 women were referred to a specialist perinatal mental health service from 04/2023-03/2024, with 66.65% (n=54,635) having a first face-to-face appointment (57). If accepted by a perinatal mental health service women are offered psychological interventions (predominantly focused on the mother) (58–60), usually delivered individually or in a remote group format by two facilitators, depending on mental health severity and safeguarding concerns. Couples therapy to reduce relationship conflict during the perinatal period is not offered consistently, if at all. Parent-infant psychotherapy referrals are made only for bonding difficulties. Given the substantial long-term negative effects of early adverse experiences (18, 55) and the capacity of positive relationships to buffer these effects (61, 62), strengthening the mother-infant-relationship is important (63). However, parenting interventions are not routinely offered in perinatal services, despite their established evidence-base in improving child and parent outcomes and reducing child maltreatment.

By examining the feasibility and acceptability of Triple P for Baby Online as a supplementary intervention, this study is the first step towards insights related to improvements in intervention access and mother and infant support in specialist perinatal services. Thus, the aims of this uncontrolled study are 1) to examine the feasibility of recruiting and engaging mothers with perinatal mental health difficulties in an online parenting intervention and retaining them in the study and 2) to explore the acceptability of this type of intervention in mothers and perinatal mental health staff. We are also interested in exploring any implementation factors associated with engagement and retention and if there is any change over time in outcome measures of maternal mental health, wellbeing, maternal self-efficacy and the perceived mother-infant bond.

## 2 Methods

### 2.1 Design

The study is an uncontrolled feasibility study, with a qualitative component exploring the intervention’s acceptability with participating mothers and staff. Indicators for psychological change over a ten-week follow-up period will be explored by pre- and post-intervention outcome measures. This uncontrolled, pre- and post-intervention design was chosen to focus on acceptability and feasibility of the intervention and to collect data to determine if the study procedures, outcome questionnaires and Treatment As Usual (TAU) as a comparative arm for a future trial are feasible.

**Figure 1:**
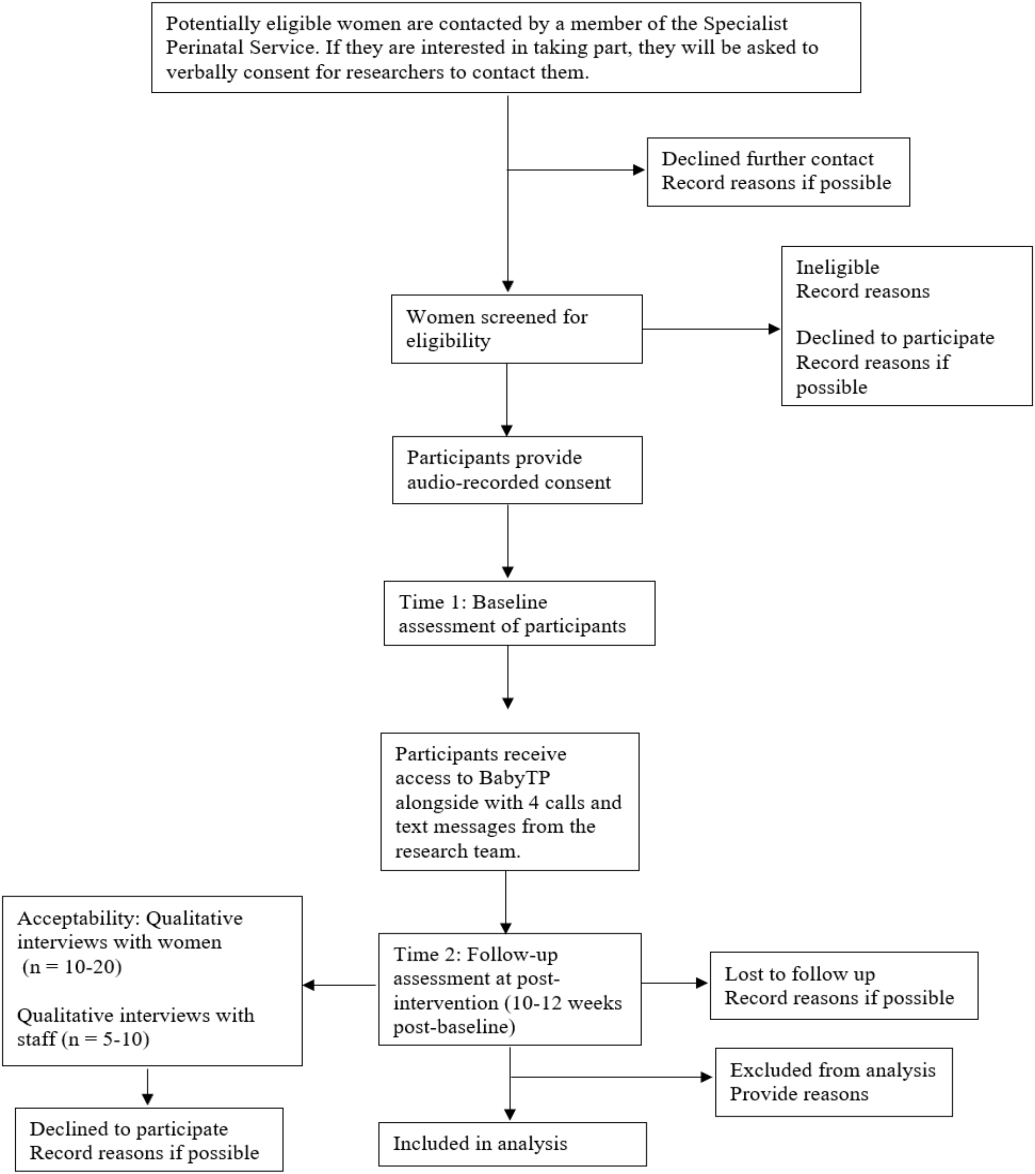
CONSORT diagram showing study design.

**Figure 1** provides an overview of the study design based on CONSORT guidelines (64). For transparency, this protocol is reported in line with the SPIRIT checklist for protocols of randomised trials (65), although any items related to randomisation and blinding (21a-24c) have been omitted from this protocol because we are describing a non-randomised trial. The TIDieR checklist for the reporting of interventions (66) has also been used (see **Supplementary Materials**).

### 2.2 Ethical approvals and research governance

The study has been approved by the National Health Service (NHS), Health Research Authority and the Greater Manchester Mental Health NHS Foundation Trust (IRAS ID: 352080). It will be conducted in accordance with the local legislation and institutional requirements. Amendments to ethical approval will be sought in the event of protocol modification. Participants will provide informed consent to participate in the study. The University of Manchester will provide oversight as the study sponsor. All staff will be trained in research governance principles (e.g., Good Clinical Practice, data protection and information governance). A Patient and Public Involvement and Engagement (PPIE) group comprising mothers with lived experience of perinatal mental health difficulties will be consulted on various aspects of the study such as content of the support calls and text messages and the topic guide. The PPIE group will convene at four scheduled meetings during the study and provide support via email between meetings. The intervention is low risk so there will not be a standard provision of ancillary and post-trial care.

### 2.3 Study setting

In this single site study, participants will be recruited from a specialist perinatal mental health community-based service, covering three large geographical areas in Greater Manchester, England, for women experiencing moderate to severe mental health problems in the perinatal period. Women referred to the service are assessed and, if eligible, assigned to a particular treatment pathway (e.g., the psychology pathway or the occupational therapy pathway). For this study, we will recruit from the psychology pathway. Mothers assigned to this pathway are typically offered individual or group specialist psychological interventions to meet their complex needs.

### 2.4 Inclusion and exclusion criteria for participants

Participants are eligible if they are a) in the third trimester of pregnancy or the mother or birthing parent of an infant aged birth to 12 months, with whom they live, or have parental responsibility for, b) aged 18 years or older, c) proficient in English to engage with the intervention and complete assessments (and take part in interviews), d) capable of giving informed consent. Participants also need to have been accepted onto the psychology pathway of the service and own or have access to a smartphone, tablet, laptop or computer to access the intervention. As Triple P for Baby Online is a supplementary intervention that participants can engage with alongside any psychological treatment, participants will not be excluded if they engage in other interventions typically offered by this service. Participants will be excluded if they are or become inpatients at the time of recruitment, because inpatient admission usually signals that additional risk factors are present (e.g., suicidal ideation or behaviours) that cannot easily be managed in a community setting.

### 2.5 Sample size considerations and recruitment

We plan to recruit a minimum of 30 participants, which is a typical and recommended sample size for feasibility studies (67, 68). As codes for the intervention are only available for 100 participants as a minimum, we could recruit up to 100 participants.

Potential mothers will be identified by perinatal mental health staff, such as the team’s clinical psychologist or assistant psychologist. Staff will consider who on their waiting lists or caseloads might be eligible and then provide them with brief information about the study in the form of an advert, or a brief study summary. Members of the research team will also attend weekly referral and allocation meetings to support staff with recruitment and potential participant identification and reduce potential selection bias. If staff have concerns about a woman’s capacity or mental state (e.g., suicidal ideation or behaviours), they would not consider that participant to be eligible.

Mothers (and birthing parents) interested in taking part will be asked for verbal consent to be contacted by a member of the study team who will then contact them to provide further information and conduct an eligibility screening. Alternatively, potential participants can self-refer to the study via a link in the study advert which staff can email to interested mothers. Potential participants will be provided with a participant information sheet and given at least 48 hours to read it. A follow-up phone call will be arranged for a mutually convenient time to answer any questions and discuss their interest in taking part. Reasons for non-participation will be collected and anonymised with potential participants’ verbal consent to inform feasibility and acceptability.

Eligible individuals who decide to participate will be enrolled to the study. A researcher will obtain their audio-recorded informed consent to take part in the study including personal identifiable information (e.g., full name, date of birth) and complete a pseudonymised electronic copy of the consent form. Identifiable information will be stored securely and separately from the study data and deleted once it is no longer needed for study purposes. The audio-recorded consent and the pseudonymised consent form will also be stored separately from one another to maximise data protection. Baseline assessment may also be conducted or started at this appointment (depending on participant preference).

Using convenience sampling, ten to 20 mothers who have participated in the study for at least seven weeks will be invited to take part in interviews to explore study and intervention acceptability qualitatively. If a large number of participants indicate interest in attending an interview, we will prioritise contacting participants with different demographic characteristics and engagement with the intervention and/or the study procedures to capture diverse views and experiences. For staff interviews, we will purposively sample five to ten staff to include a range of job roles and perinatally relevant work experience. Staff are eligible to take part if they are a healthcare professional working for this service (e.g., a clinical or counselling psychologist, CBT therapist, nursery nurse, team leader, assistant psychologist). They will be excluded if they have been in post for less than six months to ensure staff participants are familiar with the service and its service users. Separate consent to take part in an interview will be sought from mothers and staff.

### 2.6 The intervention and its delivery

In this study, participants will be offered the Triple P Positive Parenting Program for Baby (11, 30–32, 69), which aims to increase parenting skills and confidence in parents of infants (up to 12 months) by targeting the parent-baby bond, increasing the parent’s understanding of infant development, responding to the baby’s cues in a way that supports infant mental health and learning to communicate effectively with the partner/co-parent or another family member. All participants will be offered access to the self-paced Triple P for Baby Online intervention, with content offered in seven brief sessions, which are accessed online as a series of videos featuring parents discussing their experiences of various aspects of parenting in the first year and a narrator sharing factual information about infant development and parenting (see **Table 1** for details).

**Table 1:** Overview of the triple for baby online intervention structure and content.

Session number	Session title	Session content
1	Positive parenting	Provides parents with an introduction to positive parenting as an approach to raising infants, factors that have an impact on early child development, and specific parenting strategies for developing a positive relationship with their baby.
2	Understanding your baby’s development	Parents are introduced to elements that can play a role in their baby’s development and ways they can monitor and keep track of their baby’s behaviour and development.
3	Developing a positive relationship	Parents learn strategies for developing a positive relationship with their baby and how to teach their baby new skills and behaviours.
4	Responding to your baby	Parents are helped to understand crying and teach settling techniques. Parents will also learn how to apply parenting strategies to promote positive sleeping habits.
5	Survival skills	Parents are introduced to changes new parents may experience, some of the early parenting traps, and a variety of coping strategies to manage emotions (e.g., stress, anxiety, sadness).
6	Partner support	Provides parents with information on common relationship changes new parents may experience and some partner traps. The importance of communication and ideas for maintaining relationship happiness are also introduced.
7	Conclusion	Parents are encouraged to think about how to implement the skills and knowledge they have learnt during the programme in the future.

After completion of the baseline measures, the research team will provide participants with a link to register for Triple P for Baby Online. Following registration, participants will have 12 months of access to the intervention via an internet−connected device (e.g., laptop, smartphone, or tablet), at a location and time of their choice. Participants will be asked and encouraged to complete all seven sessions of the intervention within ten weeks (a timeframe chosen to account for busy perinatal periods). Participants’ care and treatment from the specialist perinatal service will continue as usual while they take part in the study; this care might include care-coordination and psychological interventions (e.g., compassion-focused therapy, cognitive behavioural therapy, eye movement desensitisation and reprocessing therapy, dialectical behaviour therapy, cognitive analytic therapy, delivered individually or as part of an online group intervention).

To support and maximise engagement and answer any questions the mother may have about the intervention, study staff will offer encouraging prompts via text messages and brief phone calls to check on their progress (prior to session 1 to help with access, then after sessions 1, 3 and 5). These phone calls will also act as ‘trouble shooting’ support for any technical or comprehension issues and they will also enable the research team to check for any adverse events.

### 2.7 Feasibility outcomes

Feasibility will be assessed by examining data related to recruitment and study retention (i.e. completion of outcome measures at follow up). To evaluate the suitability of this study to progress to a larger, randomised trial, we will use the red, amber, green (RAG) system and pre-determined progression criteria (70–72). **Table 2** provides an overview of our proposed progression criteria. We will also report on a range of feasibility indicators (e.g., number of potential participants found to be eligible, reasons for declining to participate, the extent and pattern of missing data, the number of related adverse events, etc.). In addition, the Triple P Online Management system will allow the research team to monitor participants’ progress through the programme and aggregate relevant feasibility data on engagement (e.g., how many and what sessions were completed, dates of first and last log-ins, number of log-ins per participant).

**Table 2:** Progression criteria for feasibility.

Indicator	Green	Amber	Red
Recruitment: number of eligible participants recruited within our recruitment window of 29 weeks	≥ 60% = green	50–59% = amber	≤ 49% = red
Study retention: proportion of participants completing measures at each data collection point (baseline and follow up)	≥70%	50-69%	≤49%

### 2.8 Acceptability outcomes

Intervention acceptability will be assessed quantitatively via the Client Satisfaction Questionnaire (73), administered to participants at follow up via the online survey tool, Qualtrics. Participants will be asked to answer 13 questions (rated on a 7-point Likert scale) and three open-ended questions (e.g., Do you have any other comments about the programme)?.

Qualitative exploration of acceptability will be conducted via online interviews with a sub-group of participants and staff. Acceptability in this context will focus on the exploration of attitude towards the intervention, intervention and assessment burden, and facilitators and barriers towards uptake and engagement with the intervention, as informed by Sekhon et al.’s (74) theoretical framework of acceptability.

Separate consent for this aspect of the study will be sought. Interviews will explore participants’ views of the intervention content, mode of delivery, the encouraging phone calls and text messages, and the study procedures, such as assessment burden. We will also conduct exit interviews with mothers who withdraw from the study, if possible.

### 2.9 Questionnaire outcomes

At baseline, mothers will be asked to complete the Family Background Questionnaire (75), adapted for perinatal populations, which will ask for details of their ethnicity, the age and sex of their baby, family and household composition, their marital and employment status and brief details about characteristics and experiences that may predict attrition and outcome, such as psychiatric history, educational attainment and family support. The type and duration of standard care that mothers have received while participating in the study will be recorded to report what other treatments mothers (or birthing parents) were offered and then accessed; this will test the feasibility of using Treatment As Usual (TAU) as a comparative arm for a future trial.

**Figure 2:**
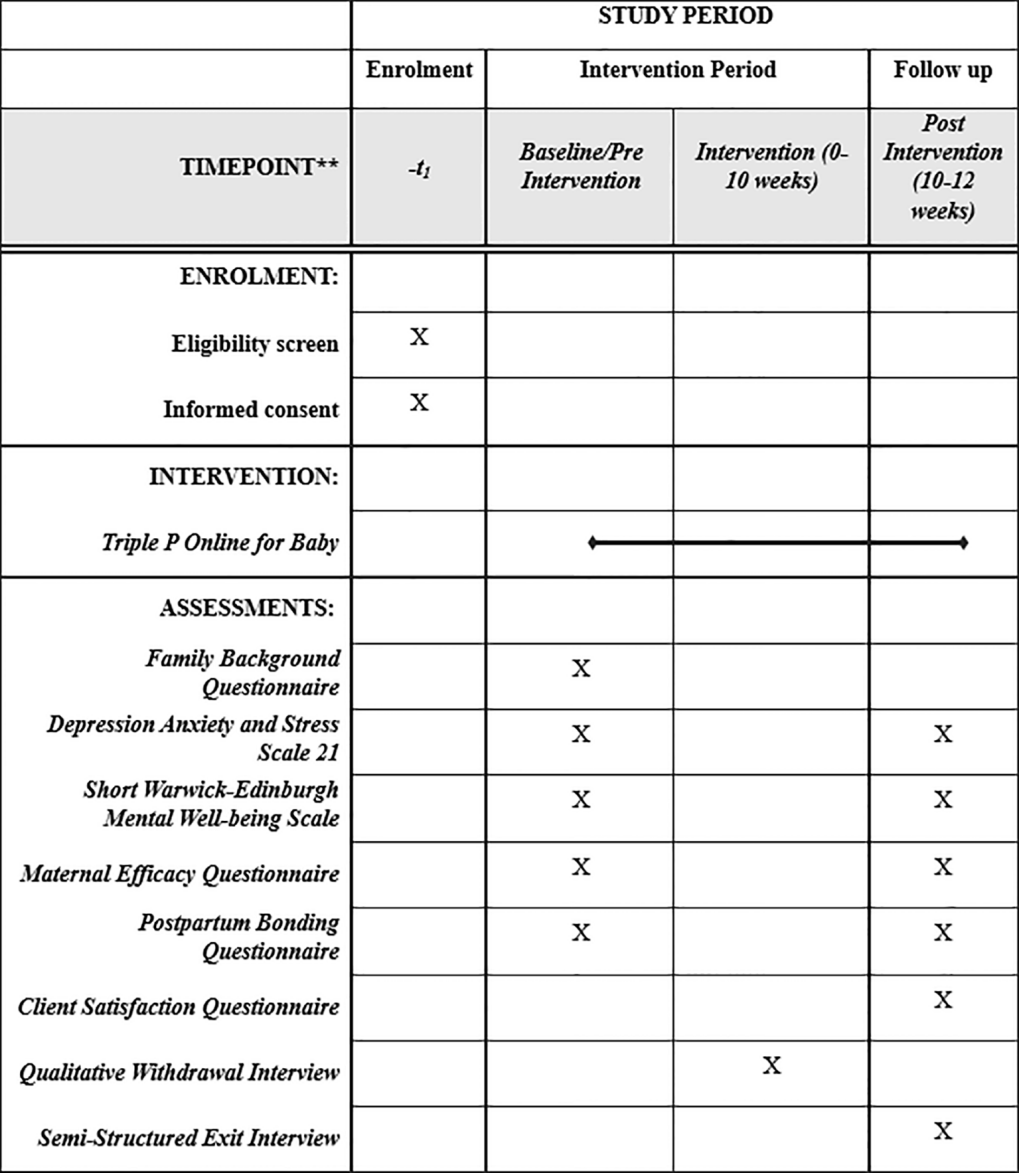
SPIRIT figure for the study.

Participants will also be asked to complete four brief validated questionnaires assessing mood (i.e., depression, anxiety, stress), wellbeing, maternal self-efficacy and the perceived mother-infant bond, chosen for their relevance, their psychometric properties and widespread use in related research (see **Table 3** for further details). They will be asked to complete these questionnaires via the online survey tool, Qualtrics, either on their own or with the support of a researcher over a telephone or video call on Microsoft Teams. At follow up (i.e., 10–12 weeks post-baseline), participants will be reminded to complete the same four questionnaires. At the same time, we will also ask them to complete the Client Satisfaction Questionnaire (73). The assessments are anticipated to take up to 30 minutes to complete. **Figure 2** provides an overview of all study data collection points.

**Table 3:** Overview of outcome questionnaires used.

Number	Outcome measure	Rationale	Item numbers and subscales	Scale type	Scoring range (minimum and maximum scores) and interpretation	Psychometric properties
1	Depression, Anxiety and Stress Scale-21 (DASS-21; 76)	To measures levels of depression, anxiety and stress	21 items: *Depression (7) *Anxiety (7) * Stress (7)	4- point Likert scale	0-21 (for each subscale) Higher the score indicates the more severe the symptoms	Cronbach’s alpha-.76-.93 Test-retest reliability- ICC = .82 -.86
2	Short Warwick Edinburgh Mental Wellbeing Scale (SWEMWBS; 77)	To measure well-being	7 items	5-point Likert scale	7-35 Scoring 7 to 35 (minimum/maximum scores) Higher scores indicate lower well-being	Cronbach’s alpha-.88-.93 Test-retest reliability- r = .86
3	Maternal Efficacy Questionnaire (MEQ; 78)^1^	To measure of parental self-efficacy	20 items: *Care taking procedures (5) *Evoking behaviours (5) *Reading behaviours (5) *Situational beliefs (5)	4-point Likert scale	20-80 (Minimum/maximum scores) Higher scores indicate higher maternal self-efficacy	Cronbach’s alpha-.91 Test-retest reliability- r= .96
4	Postpartum Bonding Questionnaire (PBQ; 79)^2^	Measures the difficulties in parent and infant bond	25 items: *Impaired bonding (PBQ-IB) (12) *Rejection and anger (PBQ-RA) (7) *Infant- focused anxiety (PBQ-IFA) (4) *Incident abuse (PBQ-LA) (2)	5-point Likert scale	0-125 (minimum/maximum) PBQ-IB: 0-60 PBQ-RA: 0-35 PBQ-IFA: 0-20 PBQ-LA: 0-10 Higher scores indicate higher perceived bonding difficulty	Cronbach’s alpha-.78-.79 Test-retest reliability- r = .77-

^1^Pregnant participants are not asked to complete this measure.

^2^An adapted version is available for pregnant women.

Participants will be encouraged and reminded to complete the follow-up questionnaires via email, text message and phone call, and asked if they would prefer to complete the measures with the assistance of a researcher. All data will be pseudonymised and entered into a database, stored on a secure server, by the research assistant and checked for accuracy and quality by the joint project managers.

### 2.10 Safety considerations

Adverse events will be monitored and reported throughout the study in line with sponsor and site reporting systems. A log of all adverse events will record all unexpected adverse events as well as reports of significant or sustained deterioration in the mental health of the participant caused by the intervention during the trial period (date of first contact to final contact). Any such deteriorations will be designated serious adverse events for the purposes of this study, even when hospitalisation is not required. Adverse events will primarily be self-reported to the research team or identified by the research team as part of their routine assessments. Enquiries about adverse events will be made by the project managers. The research team will determine if the event qualifies for expedited reporting. The intervention is low risk and we do not anticipate stopping the study; however, in the unlikely event that any serious adverse events are attributed to participation in the study, the study will be stopped.

We will inform any participant’s General Practitioner (GP) or family doctor of her participation as well as the named perinatal mental health practitioner (e.g., a Case Manager or a Clinical Psychologist) so that they are aware of the participant taking part in the study. Participants are also informed before providing consent and at the time of formal consenting that if they disclose information to the study team suggesting that either the participant or someone else is at risk of harm, the study team may have to inform authorities, such as the participant’s GP or the police. The participant will be involved in the decision as much as possible.

The research team is trained in adult and child safeguarding and in risk and distress management in perinatal mental health and parenting research contexts. The team has conducted a written risk assessment for the study and developed a Sponsor and Trust approved protocol for managing risk and distress before, during and after participant contact to ensure duty of care towards participants. If a researcher observes potential signs of mental health deterioration, the observation will be shared with the participant’s named mental health practitioner and GP. If the participant is assessed to pose an imminent risk to either herself or to someone else, the risk will be discussed with the Chief Investigator (who is also a registered Perinatal Clinical Psychologist) and, if considered appropriate, the researcher will contact emergency services.

Participants may continue to access the intervention for up to 12 months after registration if they so wish. This means participants can access supplementary aftercare in addition to usual care they are already accessing or entitled to via the perinatal mental health service.

### 2.11 Data analysis

Participant flow through the study will be reported using the CONSORT diagram (**Figure 1**). Descriptive summaries will be produced for feasibility and other outcome data (e.g., means and standard deviations or numbers and percentages, including percentages and patterns of missing data). We will use a paired t-test or Wilcoxon signed rank test for pre- and post-intervention questionnaire data and the reliable change index (80, 81) to explore changes in clinical outcomes (i.e., potential intervention benefits). However, given that this is an uncontrolled study, any results in relation to outcomes will be treated with extreme caution.

All interview data will be digitally recorded, transcribed verbatim and then analysed using Framework Analysis (82, 83). Informed by the acceptability of healthcare interventions theoretical framework (74), an initial coding framework will be developed to reflect key service and service user-determined topics covered by the interview schedule. Following data familiarisation, this framework will be augmented to encompass new emerging themes. Initially, a subset of transcripts will be coded independently by a member of the research team, and findings will be discussed by the rest of the research team and the PPIE group to develop a shared theoretical framework. This framework will then be applied to the remaining transcripts. Data will be interpreted and analysed within the final framework to structure and compare participant and staff views about the study and intervention. The trustworthiness of the final analysis will be enhanced through the integration of data from different stakeholders and through researcher triangulation.

Given the small-scale uncontrolled nature of this trial, a data monitoring committee was not deemed necessary. Instead, the research team conducted monthly trial monitoring meetings, and the project managers regularly conducted a review of trial documents.

## 3 Discussion

This will be the first study to examine the feasibility and acceptability of a self-paced parenting intervention, Triple P for Baby Online, offered to mothers in a NHS perinatal community mental health service. It will also be the first study to explore the possible benefits of this specific parenting intervention, delivered online as a self-paced programme, in mothers with moderate to severe mental health problems in the perinatal period. This study will also allow us to explore any pragmatic issues that will inform a larger trial, such as the recruitment pathway, the acceptability of the intervention and study procedures to mothers as well as perinatal mental health staff. We will identify how the intervention needs to be tailored or refined to meet the needs of this clinical group of service users in specialist perinatal community mental health settings. As it is expected that participants will present with a range of perinatal mental health problems, differences in childrearing practices and parenting concerns and different levels of parenting confidence in addressing these, this study will allow us to gauge the appropriateness of the intervention content and the practicalities of engaging with the sessions during the perinatal period, whilst under the care of a specialist perinatal mental health service.

This study will enable us to determine if progression to a full trial is indicated or not. If it is, then the current study will allow us to consider the needs for any amendments that may help recruitment and retention outcomes (e.g., the timepoint of study enrolment and intervention offer, changes to outcome measures, and content and frequency of the supportive check-in calls and text messages).

Finally, this study will also help us determine what type of training and what level of support our PPIE group members may wish to see to strengthen their involvement and support their engagement with a future trial.

The research team intends to disseminate outcomes from this study in peer-reviewed open access journals and at relevant conferences. Participants will be provided with a lay summary of the findings if they indicated that they wish to receive this information and consented to this information being emailed to them. Recruitment started in May 2025 and is ongoing. The study is set to be completed by end of February 2026.

## References

[1] Barlow J Bergman H Kornør H Wei Y Bennett C . Group-based parent training programmes for improving emotional and behavioural adjustment in young children. Cochrane Database Systematic Rev. (2016) 8:Article CD003680. doi: 10.1002/14651858.CD003680.pub3, PMID: 27478983 PMC6797064

[2] Furlong M McGilloway S Bywater T Hutchings J Smith SM Donnelly M . Behavioural and cognitive-behavioural group-based parenting programmes for early-onset conduct problems in children aged 3 to 12 years. Cochrane Database Systematic Rev. (2022) 3:Article CD008225. doi: 10.1002/14651858.CD008225.pub2, PMID: 22336837 PMC12935172

[3] Sanders MR . Triple P–Positive Parenting Program: Towards an empirically validated multilevel parenting and family support strategy for the prevention of behavior and emotional problems in children. Clin Child Family Psychol Rev. (1999) 2:71–90. doi: 10.1023/A:1021843613840, PMID: 11225933

[4] Sanders MR . Development, evaluation, and multinational dissemination of the Triple P–Positive Parenting Program. Annu Rev Clin Psychol. (2012) 8:345–79. doi: 10.1146/annurev-clinpsy-032511-143104, PMID: 22149480

[5] Sanders MR Markie-Dadds C Turner KMT . Theoretical, scientific and clinical foundations of the Triple P–Positive Parenting Program. Aust Psychol. (2003) 35:136–49. doi: 10.1080/00050060008257960, PMID: 41735180

[6] Turner KMT Sanders MR . Dissemination of evidence-based parenting and family support strategies: Learning from the Triple P–Positive Parenting Program system approach. Aggression Violent Behav. (2006) 11:176–93. doi: 10.1016/j.avb.2005.07.005, PMID: 41743167

[7] Sanders MR Kirby JN Tellegen CL Day JJ . The Triple P–Positive Parenting Program: A systematic review and meta-analysis of a multi-level system of parenting support. Clin Psychol Rev. (2014) 34:337–57. doi: 10.1016/j.cpr.2014.04.003, PMID: 24842549

[8] Li N Peng J Li Y . Effects and moderators of the Triple P–Positive Parenting Program on children’s social, emotional, and behavioral problems: Systematic review and meta-analysis. Front Psychol. (2021) 12:719145. doi: 10.3389/fpsyg.2021.719145, PMID: 34512467 PMC8427298

[9] Nieminen P Sourander A Helstelä L . Long-term effects (2–4 years) of the Triple P program on parental stress, self-efficacy, and parent–child relationship quality in a community setting. J Child Family Stud. (2023) 32:1528–42. doi: 10.1007/s10826-023-02663-4, PMID: 41746348

[10] Pontoppidan M Klest SK Patras J Rayce SB . Effects of universally offered parenting interventions for parents with infants: A systematic review. BMJ Open. (2016) 6:e011706. doi: 10.1136/bmjopen-2016-011706, PMID: 27683513 PMC5051433

[11] Sanders MR . The Triple P system of evidence-based parenting support: Past, present, and future directions. Clin Child Family Psychol Rev. (2023) 26:880–903. doi: 10.1007/s10567-023-00441-8, PMID: 37432507 PMC10640495

[12] de Graaf I Speetjens P Smit F de Wolff M Tavecchio L . Effectiveness of the Triple P Positive Parenting Program on parenting: A meta-analysis. Family Relations. (2008) 57:553–66. doi: 10.1111/j.1741-3729.2008.00522.x, PMID: 18475003

[13] Nowak C Heinrichs N . A comprehensive meta-analysis of Triple P–Positive Parenting Program using hierarchical linear modeling: Effectiveness and moderating variables. Clin Child Family Psychol Rev. (2008) 11:114–44. doi: 10.1007/s10567-008-0033-0, PMID: 18509758

[14] Cowan CP Cowan PA . When partners become parents: The big life change for couples. New York: Lawrence Erlbaum (2000).

[15] Slade A . Parental reflective functioning and the development of attachment relationships. Psychoanalytic Inq. (2018) 38:212–29. doi: 10.1080/07351690.2018.1476802, PMID: 41735180

[16] Woolhouse H Gartland D Hegarty K Donath S Brown S . Depressive symptoms and intimate partner violence in the 12 months after childbirth: A prospective pregnancy cohort study. BJOG: Int J Obstetrics Gynaecol. (2014) 121:772–9. doi: 10.1111/1471-0528.12581, PMID: 22145631

[17] O’Hara MW McCabe JE . Postpartum depression: Current status and future directions. Annu Rev Clin Psychol. (2013) 9:379–407. doi: 10.1146/annurev-clinpsy-050212-185612, PMID: 23394227

[18] Stein A Malmberg LE Leach P Barnes J Sylva K FCCC Team . The influence of different forms of early childcare on children’s emotional and behavioural development at school entry. Child: Care Health Dev. (2013) 39:676–87. doi: 10.1111/j.1365-2214.2012.01421.x, PMID: 22928988

[19] Risi A Pickard J Bird AL . The implications of parent mental health and wellbeing for parent–child attachment: A systematic review. PloS One. (2021) 16:e0260891. doi: 10.1371/journal.pone.0260891, PMID: 34914730 PMC8675743

[20] Shonkoff JP Phillips D . From neurons to neighborhoods: The science of early childhood development. Washington, D. C.: National Academy Press (2000). 25077268

[21] Feldman R . Mutual influences between child emotion regulation and parent–child reciprocity support development across the first 10 years of life. Dev Sci. (2015) 18:281–91. doi: 10.1111/desc.12224, PMID: 26439059

[22] Booth A Macdonald JA Youssef GJ . Contextual stress and maternal sensitivity: A meta-analytic review of stress associations with the Maternal Behavior Q-Sort in observational studies. Dev Rev. (2018) 48:145–77. doi: 10.1016/j.dr.2018.02.002, PMID: 41743167

[23] Ainsworth MDS Blehar M Waters E Wall S . Patterns of attachment: A psychological study of the strange situation. New York: Lawrence Erlbaum (1978).

[24] Sroufe LA . Attachment and development: A prospective, longitudinal study from birth to adulthood. Attachment Hum Dev. (2005) 7:349–67. doi: 10.1080/14616730500365928, PMID: 16332580

[25] de Cock E Prior E Wilson E . Partner support during pregnancy and maternal–infant bonding: Evidence from a longitudinal perinatal sample. Infant Behav Dev. (2023) 72:101871. doi: 10.1016/j.infbeh.2023.101871, PMID: 37544195

[26] Center on the Developing Child . From best practices to breakthrough impacts: A science-based approach to building a more promising future for young children and families. Cambridge, MA: Harvard University (2016). Available online at: https://developingchild.harvard.edu/.

[27] Leadsom A Field F Burstow P Lucas C . The 1001 critical days: The importance of the conception to age two period. Wave Trust (2013). Available online at: https://www.wavetrust.org/1001-critical-days-the-importance-of-the-conception-to-age-two-period.

[28] Public Health England . Health matters: Giving every child the best start in life. London: UK Government (2016). Available online at: https://www.gov.uk/government/publications/health-matters-giving-every-child-the-best-start-in-life/health-matters-giving-every-child-the-best-start-in-life.

[29] National Health Service . The NHS long term plan. National Archives (2019). Available online at: https://webarchive.nationalarchives.gov.uk/ukgwa/20230418155402/https:/www.longtermplan.nhs.uk/publication/nhs-long-term-plan/.

[30] Spry C Morawska A Sanders MR . Baby Triple P group workbook. Brisbane: Triple P International Pty Ltd (2011).

[31] Spry CHM . *The Baby Triple P project: Effects of a parenting intervention to promote a successful transition to parenthood*. Brisbane, Australia: University of Queensland (2013).

[32] Morawska A Herd M Jackson C . Triple P for Baby: Parenting support across the perinatal period. Int J Birth Parent Educ. (2023) 10:31–7.

[33] Adina J Morawska A Mitchell AE Haslam D Ayuku D . Enhancing parenting skills for pregnant women with depressive symptoms: A randomised controlled trial of Triple P for Baby in Kenya. Behav Res Ther. (2025) 195:Article 104886. doi: 10.1016/j.brat.2025.104886, PMID: 41092685

[34] Colditz PB Boyd RN Winter L Pritchard M Gray PH Whittingham K . A randomized trial of Baby Triple P for preterm infants: Child outcomes at 2 years of corrected age. J Pediatr. (2019) 210:48–54.e2. doi: 10.1016/j.jpeds.2019.01.024, PMID: 30857773

[35] Ferrari AJ Whittingham K Boyd R Sanders M Colditz P . Prem Baby Triple P: A new parenting intervention for parents of infants born very preterm: Acceptability and barriers. Infant Behav Dev. (2011) 34:602–9. doi: 10.1016/j.infbeh.2011.06.004, PMID: 21798599

[36] Evans T Boyd RN Colditz P Sanders M Whittingham K . Mother–very preterm infant relationship quality: RCT of Baby Triple P. J Child Family Stud. (2017) 26:284–95. doi: 10.1007/s10826-016-0555-x, PMID: 41746348

[37] Henderson M Wittkowski A Buston K Crawford K MacLachlan A McConnachie A . Evaluation of parenting interventions for those with additional health and social care needs during pregnancy: THRIVE, a multi-arm RCT with embedded economic and process components. Public Health Res. (2025) 13:1–138. doi: 10.3310/KYMT5407, PMID: 40351091

[38] McPherson K Wiseman K Jasilek A McAloney-Kocaman K Morawska A Haig C . Baby Triple P: A randomized controlled trial testing the efficacy in first-time parent couples. J Child Family Stud. (2022) 31:2156–74. doi: 10.1007/s10826-022-02345-7, PMID: 41746348

[39] Mihelic M Morawska A Filus A . Does a perinatal parenting intervention work for fathers? A randomized controlled trial. Infant Ment Health J. (2018) 39:687–98. doi: 10.1002/imhj.21748, PMID: 30339721

[40] Popp L Fuths S Schneider S . The relevance of infant outcome measures: A pilot RCT comparing Baby Triple P Positive Parenting Program with care as usual. Front Psychol. (2019) 10:2425. doi: 10.3389/fpsyg.2019.02425, PMID: 31736826 PMC6828945

[41] Seah KF . *Enhancing sensitive parenting and reducing parenting stress: effects of Baby Triple P as a postnatal parenting intervention*. Brisbane, Australia: School of Psychology, The University of Queensland (2016). doi: 10.14264/uql.2016.328

[42] Tsivos Z-L Calam R Sanders MR Wittkowski A . A pilot randomised controlled trial to evaluate the feasibility and acceptability of the Baby Triple P Positive Parenting Programme in mothers with postnatal depression. Clin Child Psychol Psychiatry. (2015) 20:532–54. doi: 10.1177/1359104514531589, PMID: 24778436 PMC4591516

[43] O’Brien R Buston K Wight D McGee E White J Henderson M . A realist process evaluation of Enhanced Triple P for Baby and Mellow Bumps, within a Trial of Health Relationship Initiatives from the Very Early years (THRIVE): study protocol for a randomized controlled trial. Trials. (2019) 20:351. doi: 10.1186/s13063-019-3395-3, PMID: 31196169 PMC6567913

[44] Wittkowski A Emsley R Bee PE Camacho E Calam R Abel KM . A feasibility randomized controlled trial of a parenting intervention offered to women with severe mental health problems and delivered in a Mother and Baby Unit setting: The IMAgINE Study outcomes. Front Psychiatry. (2022) 13:815018. doi: 10.3389/fpsyt.2022.815018, PMID: 35651824 PMC9149174

[45] Sanders MR Dittman CK Farruggia SP Keown LJ . A comparison of online versus workbook delivery of a self-help positive parenting program. J Primary Prev. (2014) 35:125–33. doi: 10.1007/s10935-014-0339-2, PMID: 24500106

[46] Triple P UK Ltd . Written evidence submitted by Triple P UK (NFS0010). London: UK Parliament (2024). Available online at: https://committees.parliament.uk/writtenevidence/131291/pdf/.

[47] Whittle K Protheroe N . Triple P for Baby: Manager implementation planning session. Triple P UK (2023).

[48] Department for Work and Pensions . Reducing Parental Conflict Programme 2018–2022: An evaluation of the effects of interventions on parental relationships and children. London: UK Government (2023). Available online at: https://www.gov.uk/government/publications/reducing-parental-conflict-programme-2018-to-2022-an-evaluation-of-the-effects-of-interventions/reducing-parental-conflict-programme-2018-2022-an-evaluation-of-the-effects-of-interventions-on-parental-relationships-and-children.

[49] Department for Education, & Department for Levelling Up, Housing & Communities . Chapter 3: the national supporting families outcome framework. In: . UK Government. Supporting Families programme guidance 2022 to 2025. (2022). Available online at: https://www.gov.uk/government/publications/supporting-families-programme-guidance-2022-to-2025/chapter-3-the-national-supporting-families-outcome-framework.

[50] National Health Service England . Perinatal mental health. Available online at: https://www.england.nhs.uk/mental-health/perinatal/ (Accessed February 11, 2026).

[51] Felker A Patel R Kotnis R Kenyon S Knight M . Saving lives, improving mothers’ care: Lessons learned to inform maternity care from the UK and Ireland confidential enquiries into maternal deaths and morbidity 2021–23 (MBRRACE-UK Maternal Report 2025). Oxford: National Perinatal Epidemiology Unit, University of Oxford (2025).

[52] Easter A De Backer K Fisher L Slade P Bridle L Challacombe F . ESMI-III: The effectiveness and implementation of maternal mental health services interim report: Phase 1. NIHR Applied Research Collaboration South London (2022). Available online at: https://arc-sl.nihr.ac.uk/sites/default/files/uploads/files/ESMII-report-august-2022_final%20(1).pdf.

[53] National Health Service England . The five year forward view for mental health (2016). Available online at: https://www.england.nhs.uk/wp-content/uploads/2016/02/Mental-Health-Taskforce-FYFV-final.pdf (Accessed February 11, 2026).

[54] Gentile S . Untreated depression during pregnancy: Short- and long-term effects in offspring. A systematic review. Neuroscience. (2017) 342:154–66. doi: 10.1016/j.neuroscience.2015.09.001, PMID: 26343292

[55] Junge C Garthus-Niegel S Slinning K Polte C Simonsen TB Eberhard-Gran M . The impact of perinatal depression on children’s social–emotional development: A longitudinal study. Maternal Child Health J. (2017) 21:607–15. doi: 10.1007/s10995-016-2146-, PMID: 27485491

[56] National Institute for Health and Care Excellence (NICE) . Antenatal and postnatal mental health: Clinical management and service guidance (CG192) (2020). Available online at: https://www.nice.org.uk/guidance/cg192 (Accessed February 11, 2026). 31990493

[57] National Health Service England Digital . Mental health bulletin: 2023–2024 annual report (2024). Available online at: https://digital.nhs.uk/data-and-information/publications/statistical/mental-health-bulletin/2023-24-annual-report (Accessed February 11, 2026).

[58] Division of Clinical Psychology . Briefing Paper 8: Perinatal service provision: The role of perinatal clinical psychology. Leicester: British Psychological Society (2016).

[59] O’Brien J Gregg L Wittkowski A . Finding my voice again”: Women’s experiences of psychological therapy in perinatal secondary care settings—A qualitative study. Front Psychiatry. (2024) 15:1240855. doi: 10.3389/fpsyt.2024.1240855, PMID: 38863602 PMC11165924

[60] Royal College of Psychiatrists . CR197: Perinatal mental health services—Recommendations for the provision of services for childbearing women. London: Royal College of Psychiatrists. (2015).

[61] National Institute for Health and Care Excellence (NICE) . PH40: Social and emotional wellbeing in early years (2012). Available online at: https://www.nice.org.uk/guidance/ph40/resources/social-and-emotional-wellbeing-early-years-pdf-1996351221445 (Accessed February 11, 2026).

[62] Marmot Review Team . The Marmot Review: Fair society, healthy lives. Institute of Health Equity (2010). Available online at: https://www.instituteofhealthequity.org/resources-reports/fair-society-healthy-lives-the-marmot-review/fair-society-healthy-lives-full-report-pdf.pdf.

[63] Balbernie R . The importance of secure attachment for infant work for perinatal mental health services. J Health Visiting. (2013) 1:210–7. doi: 10.12968/johv.2013.1.4.210, PMID: 36641652

[64] Hopewell S Chan AW Collins GS Hróbjartsson A Moher D Schulz KF . CONSORT 2025 statement: Updated guideline for reporting randomised trials. BMJ. (2025) 388:e081123. doi: 10.1136/bmj-2024-081123, PMID: 40228833 PMC11995449

[65] Chan AW Boutron I Hopewell S Moher D Schulz KF Collins GS . SPIRIT 2025 statement: Updated guideline for protocols of randomised trials. BMJ. (2025) 389:e081477. doi: 10.1136/bmj-2024-081477, PMID: 40294953 PMC12035670

[66] Hoffmann TC Glasziou PP Boutron I Milne R Perera R Moher D . Better reporting of interventions: Template for intervention description and replication (TIDieR) checklist and guide. BMJ. (2014) 348:g1687. doi: 10.1136/bmj.g1687, PMID: 24609605

[67] Lancaster GA Dodd S Williamson PR . Design and analysis of pilot studies: Recommendations for good practice. J Eval Clin Pract. (2004) 10:307–12. doi: 10.1111/j.2002.384.doc.x, PMID: 15189396

[68] Totton N Lin J Julious S Chowdhury M Brand A . A review of sample sizes for UK pilot and feasibility studies on the ISRCTN registry from 2013 to 2020. Pilot Feasibility Stud. (2023) 9:188. doi: 10.1186/s40814-023-01416-w, PMID: 37990337 PMC10662929

[69] Triple P UK Ltd . Triple P online for baby (2025). Available online at: https://www.triplep-parenting.uk.net/uk/parenting-courses/triple-p-online-for-baby/ (Accessed February 11, 2026).

[70] Avery KNL Williamson PR Gamble C O’Connell Francischetto E Metcalfe C Davidson P . Informing efficient randomised controlled trials: Exploration of challenges in developing progression criteria for internal pilot studies. BMJ Open. (2017) 7:e013537. doi: 10.1136/bmjopen-2016-013537, PMID: 28213598 PMC5318608

[71] Lewis M Bromley K Sutton CJ McCray G Myers HL Lancaster GA . Determining sample size for progression criteria for pragmatic pilot RCTs: The hypothesis test strikes back! Pilot Feasibility Stud. (2021) 7:40. doi: 10.1186/s40814-021-00770-x, PMID: 33536076 PMC7856754

[72] Nielsen D d’Apice K Cheung RW Bryant M Heald R Storr C . A randomised controlled feasibility trial of an early years language development intervention: Results of the “outcomes of Talking Together evaluation and results” (oTTer) project. Pilot Feasibility Stud. (2023) 9:107. doi: 10.1186/s40814-023-01333-y, PMID: 37386614 PMC10308722

[73] Sanders MR Markie-Dadds C Turner KMT . Practitioner’s manual for standard triple P. Brisbane: Triple P International Pty Ltd (2001).

[74] Sekhon M Cartwright M Francis JJ . Acceptability of healthcare interventions: An overview of reviews and development of a theoretical framework. BMC Health Serv Res. (2017) 17:88. doi: 10.1186/s12913-017-2031-8, PMID: 28126032 PMC5267473

[75] Sanders MR Morawska A . Family Background Questionnaire. Brisbane: Parenting and Family Support Centre (2010).

[76] Henry JD Crawford JR . The short-form version of the depression anxiety stress scale (DASS-21): Construct validity and normative data in a large non-clinical sample. British Journal of Clinical Psychology. (2009) 44:227–39. 10.1348/014466505X29657 16004657

[77] Stewart-Brown S Tennant A Tennant R . Internal construct validity of the Warwick-Edinburgh Mental Well-being Scale (WEMWBS): a Rasch analysis using data from the Scottish Health Education Population Survey. Health and Quality of Life Outcomes. (2009) 7:15. doi:10.1186/1477-7525-7-15. PMID: 19228398 PMC2669062

[78] Teti DM Gelfand DM . Behavioral competence among mothers of infants in the first year: The mediational role of maternal self-efficacy. Child Development. (1991) 62(5):918–29. 10.1111/j.1467-8624.1991.tb01580.x 1756667

[79] Brockington I Oates J George S Turner D Vostanis P Sullivan M . A screening questionnaire for mother-infant bonding disorders. Archives of Women’s Mental Health. (2001) 3:133–40.

[80] Jacobson NS Follette WC Revenstorf D . Psychotherapy outcome research: Methods for reporting variability and evaluating clinical significance. Behav Ther. (1984) 15:336–52. doi: 10.1016/S0005-7894(84)80002-7, PMID: 41635714

[81] Jacobson NS Truax P . Clinical significance: A statistical approach to defining meaningful change in psychotherapy research. J Consulting Clin Psychol. (1991) 59:12–9. doi: 10.1037/0022-006X.59.1.12, PMID: 2002127

[82] Gale NK Heath G Cameron E Rashid S Redwood S . Using the framework method for the analysis of qualitative data in multi-disciplinary health research. BMC Med Res Method. (2013) 13:117. doi: 10.1186/1471-2288-13-117, PMID: 24047204 PMC3848812

[83] Ritchie J Spencer L . Qualitative data analysis for applied policy research. In: Bryman A Burgess RG , editors. Analyzing qualitative data. London: Routledge (1994). p. 173–94.

