# Supplementary material for: The Baby Triple P online positive parenting programme for mothers accessing community perinatal mental health care (the OPAL study): a feasibility study protocol
Source: Front Psychiatry. 2026 Mar 3;17:1766060. doi: 10.3389/fpsyt.2026.1766060 (PMC12992284; doi:10.3389/fpsyt.2026.1766060)
Supplement: Supplementary file 2 [file Table2.docx]

**Supplementary Table 2: The TIDieR (Template for Intervention Description and Replication) Checklist**

| **Item number** | | **Item** | **Location of information** |
| --- | --- | --- | --- |
|  | **BRIEF NAME** | |  |
| **1.** | Provide the name or a phrase that describes the intervention. | | 2.6 The intervention and its delivery |
|  | **WHY** | |  |
| **2.** | Describe any rationale, theory, or goal of the elements essential to the intervention. | | 1. Introduction |
|  | **WHAT** | |  |
| **3.** | Materials: Describe any physical or informational materials used in the intervention, including those provided to participants or used in intervention delivery or in training of intervention providers. Provide information on where the materials can be accessed (e.g. online appendix, URL). | | 2.6 The intervention and its delivery |
| **4.** | Procedures: Describe each of the procedures, activities, and/or processes used in the intervention, including any enabling or support activities. | | 2.6 The intervention and its delivery |
|  | **WHO PROVIDED** | |  |
| **5.** | For each category of intervention provider (e.g. psychologist, nursing assistant), describe their expertise, background and any specific training given. | | N/A (self-directed intervention) |
|  | **HOW** | |  |
| **6.** | Describe the modes of delivery (e.g. face-to-face or by some other mechanism, such as internet or telephone) of the intervention and whether it was provided individually or in a group. | | 2.6. The intervention and its delivery |
|  | **WHERE** | |  |
| **7.** | Describe the type(s) of location(s) where the intervention occurred, including any necessary infrastructure or relevant features. | | N/A (intervention accessed at a place of the participant’s choosing) |
|  | **WHEN and HOW MUCH** | |  |
| **8.** | Describe the number of times the intervention was delivered and over what period of time including the number of sessions, their schedule, and their duration, intensity or dose. | | 2.6 The intervention and its delivery |
|  | **TAILORING** | |  |
| **9.** | If the intervention was planned to be personalised, titrated or adapted, then describe what, why, when, and how. | | N/A |
|  | **MODIFICATIONS** | |  |
| **10.** | If the intervention was modified during the course of the study, describe the changes (what, why, when, and how). | | N/A |
|  | **HOW WELL** | |  |
| **11.** | Planned: If intervention adherence or fidelity was assessed, describe how and by whom, and if any strategies were used to maintain or improve fidelity, describe them. | | 2.6. The intervention and its delivery |
| **12.** | Actual: If intervention adherence or fidelity was assessed, describe the extent to which the intervention was delivered as planned. | | N/A |
